# Recent Progress of Imprinted Polymer Photonic Waveguide Devices and Applications

**DOI:** 10.3390/polym10060603

**Published:** 2018-05-31

**Authors:** Xiu-You Han, Zhen-Lin Wu, Si-Cheng Yang, Fang-Fang Shen, Yu-Xin Liang, Ling-Hua Wang, Jin-Yan Wang, Jun Ren, Ling-Yun Jia, Hua Zhang, Shu-Hui Bo, Geert Morthier, Ming-Shan Zhao

**Affiliations:** 1 School of Optoelectronic Engineering and Instrumentation Science, Dalian University of Technology, Dalian 116024, China; zhenlinwu@dlut.edu.cn (Z.-L.W.); ysc_@mail.dlut.edu.cn (S.-C.Y.); maili@mail.dlut.edu.cn (F.-F.S.); liangyuxin0318@mail.dlut.edu.cn (Y.-X.L.); 2 Photonics Research Group, Department of Information Technology (INTEC), Ghent University-IMEC, 9000 Ghent, Belgium; geert.morthier@intec.ugent.be; 3 College of Physics and Information Engineering, Fuzhou University, Fuzhou 350116, China; linghua.wang@fzu.edu.cn; 4 School of Chemical Engineering, Dalian University of Technology, Dalian 116024, China; wangjinyan@dlut.edu.cn; 5 School of Life Science and Biotechnology, Dalian University of Technology, Dalian 116024, China; renjun@mail.dlut.edu.cn (J.R.); lyj81@dlut.edu.cn (L.-Y.J.); 6 Key Laboratory of Photochemical Conversion and Optoelectronic Materials, Technical Institute of Physics and Chemistry, Chinese Academy of Sciences, Beijing 100190, China; zhanghua@mail.ipc.ac.cn (H.Z.); boshuhui@mail.ipc.ac.cn (S.-H.B.)

**Keywords:** polymer photonics, imprinting technique, passive photonic integrated waveguide devices, active photonic integrated waveguide devices, photonic biosensors

## Abstract

Polymers are promising materials for fabricating photonic integrated waveguide devices. Versatile functional devices can be manufactured using a simple process, with low cost and potential mass-manufacturing. This paper reviews the recent progress of polymer photonic integrated devices fabricated using the UV imprinting technique. The passive polymer waveguide devices for wavelength filtering, power splitting, and light collecting, and the active polymer waveguide devices based on the thermal-optic tuning effect, are introduced. Then, the electro-optic (EO) modulators, by virtue of the high EO coefficient of polymers, are described. Finally, the photonic biosensors, which are based on low-cost and biocompatible polymer platforms, are presented.

## 1. Introduction

Polymers, which have the advantages of low cost, flexile tuning of their refractive index, and a designable function with synthetization at the molecular level, have promising applications for fabricating photonic integrated waveguide devices [1,2,3,4,5]. Showing the high thermal-optic (TO) and electro-optic (EO) coefficients, they are excellent materials for low-power TO functional devices and low half-wave voltage, high-bandwidth EO modulators [6,7,8]. With the direct physical adsorption of proteins on the waveguide surface or their immobilization in a covalent way, they are also desirable materials for label-free optical biosensors [9,10,11,12]. In addition, due to their good compatibility on diverse substrates, they provide good platforms for the hybrid integration of photonic chips [13,14,15]. 

Besides the conventional photolithography and etching technique, the imprinting technique can be utilized to fabricate polymer photonic waveguide devices, which can fully exert the plastic property of polymer materials. The imprinting technique was initiated by S.Y. Chou and co-workers in 1990s to fabricate nanometer structures to overcome the diffraction limitation of photolithography [16,17]. It involved a modification of the manufacturing process and was similar to the nanofabricating process developed at NTT Laboratories in Japan in 1970s [18] and by Wulff’s and the Mosbach’s groups in 1980s [19,20]. The technique has been being widely investigated, not only in academic institutes but also in the industrial community [21,22]. Several papers have been presented to review the progress of the imprinting technique comprehensively, including the imprinting craft, the material requirement, and the fabricated function components [23,24,25,26,27,28,29,30,31,32,33]. 

Since the nanofabricating process was developed at NTT Laboratories, which is considered as the rudiment of the imprinting technique, it has been investigated for over 40 years. Several commercial polymer materials are available for imprinting craft. For thermal imprinting, there are Poly (methyl methacrylate) PMMA, Polystyrene (PS), Polycarbonate (PC), and Cyclic olefin copolymer (COC). For UV imprinting, there is a UV15 series from Masterbond Inc., Ormostamp and Ormocore from Micro Resist Technology Inc., and an NOA series from Norland Inc. Also, for the combined thermal and UV imprinting, there is a SU8 series from MicroChem Corp. and an MR series from Micro Resist Technology. Certainly, there are still some critical issues to be solved to obtain good performance imprinted structures, elements, and devices. First, the imprinting technique is a contact process. The pattern distortions can easily happen during the mold separation process. The flexible/soft molds can be applied, or the mold surface can be coated with a thin anti-adhesive film to facilitate the demolding process. Second, there is usually a residual layer left on the substrate after imprinting, which needs to be removed before subsequent processing. It can be removed by using the etching process, or it can be decreased by using improved techniques, such as the selective imprint technique.

Photonic integrated waveguide devices have broad applications in optical fiber communication systems, the photonic on-chip interconnection network, optical biosensors, and the microwave photonic signal processing system. In this paper, the recent progress of polymer photonic waveguide devices fabricated using the imprinting technique, and their applications are reviewed. In order to understand the development of imprinted polymer waveguide devices, some works conducted at the beginning of the imprinting technique are also cited. The rest of the paper is organized as follows. Section 2 introduces briefly the imprinting techniques and focuses on the UV imprinting technique. Section 3 presents the passive photonic integrated devices based on the imprinted polymer waveguide, including microring resonators, optical waveguide splitters, arrayed waveguide gratings, long-period waveguide gratings, and microlenses. Section 4 reviews the active photonic integrated devices using imprinting techniques, for example, TO tuning filters, optical switches, and variable optical attenuators. Section 6 presents the polymeric EO materials and the imprinted EO modulators (EOMs). In Section 5, the optical biosensors based on imprinted polymer waveguides, such as microring resonators and Young’s interferometers, are introduced. Finally, the conclusions and outlook are given in Section 7. 

## 2. Imprinting Techniques

**Figure 1:**
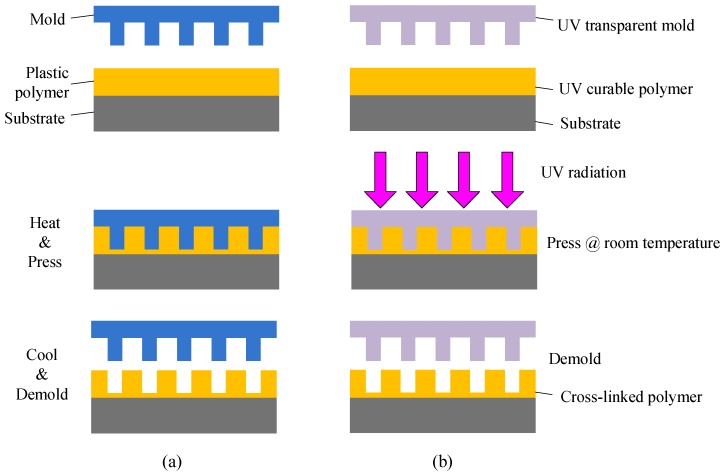
Schematic of fabrication process: (**a**) thermal imprinting and (**b**) UV imprinting.

To overcome the diffraction limitation of photolithography, the imprinting technique was initiated by S.Y. Chou and co-workers in 1990s [16,17]. Ever since the nanometer scale structures were patterned using the imprinting technique, it has been considered as a promising alternative to expensive UV, deep-UV, and extreme-UV optical lithography for semiconductor integrated circuit industry [21]. Certainly, not only the nanometer scale structures, but also various dimensions, ranging from the nanometer to the micrometer or even millimeter scale, can be formed using the imprinting technique. Therefore, besides the integrated circuits, the photonic and optoelectronic devices can be fabricated using the highly efficient and inexpensive imprinting technique [27]. By using the master mold (hard mold or soft mold) fabricated by photolithography (for large-scale features) or electron-beam lithography (for small-scale features), a reverse replica of the mold pattern can be formed on the polymer layer through heating and applied pressure for solid plastic polymers or pressing and following UV curation for liquid polymers, as shown in Figure 1a,b, which is known as the thermal imprinting technique and the UV imprinting technique [24], respectively.

The UV imprinting technique, compared to the thermal imprinting method, has several advantages. (I) The UV imprinting process works at room temperature, in most cases under low pressure vacuum contact. This is favorable for molding polymer films onto delicate or curved substrates. The mechanical properties of the mold are also less stringent, and such molds can be obtained economically by replicating the master mold; (II) The UV curing process is rapid, which can be as short as tens of seconds. Therefore, high throughput can be achieved with simple equipment and easy processing; (III) The final properties of the imprinted structures can be finely tuned by the mix ratio of components in polymer materials or the UV irradiation time. For example, high aspect ratio structures can be obtained from polymeric precursors with lower viscosity.

For the UV imprinting technique, the mold should be transparent for UV irradiation. There are two kinds of UV transparent molds. One is the hard mold made of the dielectrics, such as silicon oxide and silicon nitride [34], and the other is the soft mold made of polymer materials, such as polydimethylsiloxane (PDMS) [35,36,37], perfluoropolyether (PFPE) [38,39], and UV-curable resins [40]. The soft mold is relatively cheap due to the simple fabrication process. In addition, it is flexible in terms of the geometry and curvature of the surface, which provides better contact with the substrate. Furthermore, the demolding is usually easy using the soft mold, especially for large-area imprinting [41,42]. Therefore, the UV imprinting technique with soft mold, named the UV soft imprinting technique, is being widely investigated and applied to fabricate photonic integrated devices.

## 3. Passive Photonic Integrated Waveguide Devices

The passive photonic integrated waveguide devices, such as microring resonators, waveguide splitters, arrayed waveguide gratings, long-period gratings, and microlenses, are key components consisting of large scale integration photonic circuits. This section presents recent work on passive photonic integrated waveguide devices using the UV imprinting technique.

### 3.1. Microring Resonators

**Figure 2:**
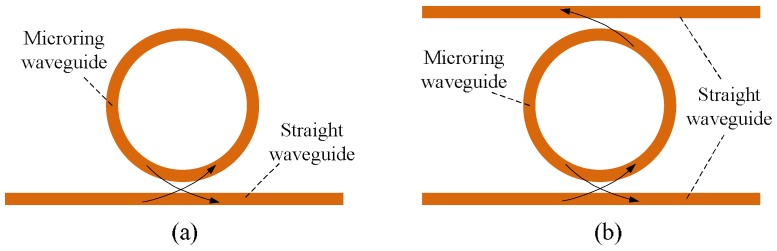
Schematic structures of microring resonators. (**a**) “All-pass” type; (**b**) “add-drop” type.

The basic microring resonator usually consists of a microring waveguide coupled with a single straight waveguide or double straight waveguides, as shown in Figure 2a,b. They can be utilized as the key structures of filters, modulators, switches, and sensors in the photonic integrated circuit [43]. 

For the imprinted polymer waveguide, the residual layer of waveguide core should be thin enough to confine the optical mode field in the core section. Otherwise, it results in a large radiation loss due to the expanding of the optical field into the residual layer, especially for the bent waveguide. The etching process is generally needed to reduce the thickness of the residual layer [44]. However, it increases the complexity of waveguide fabrication. Two new kinds of imprinted polymer waveguide structures have been developed. One is the inverted ridge waveguide generated by imprinting the cladding, and the other is the ridge waveguide with a thin residual layer generated using the selective imprinting technique.

#### 3.1.1. Microring Resonators with Inverted Ridge Waveguide

**Figure 3:**
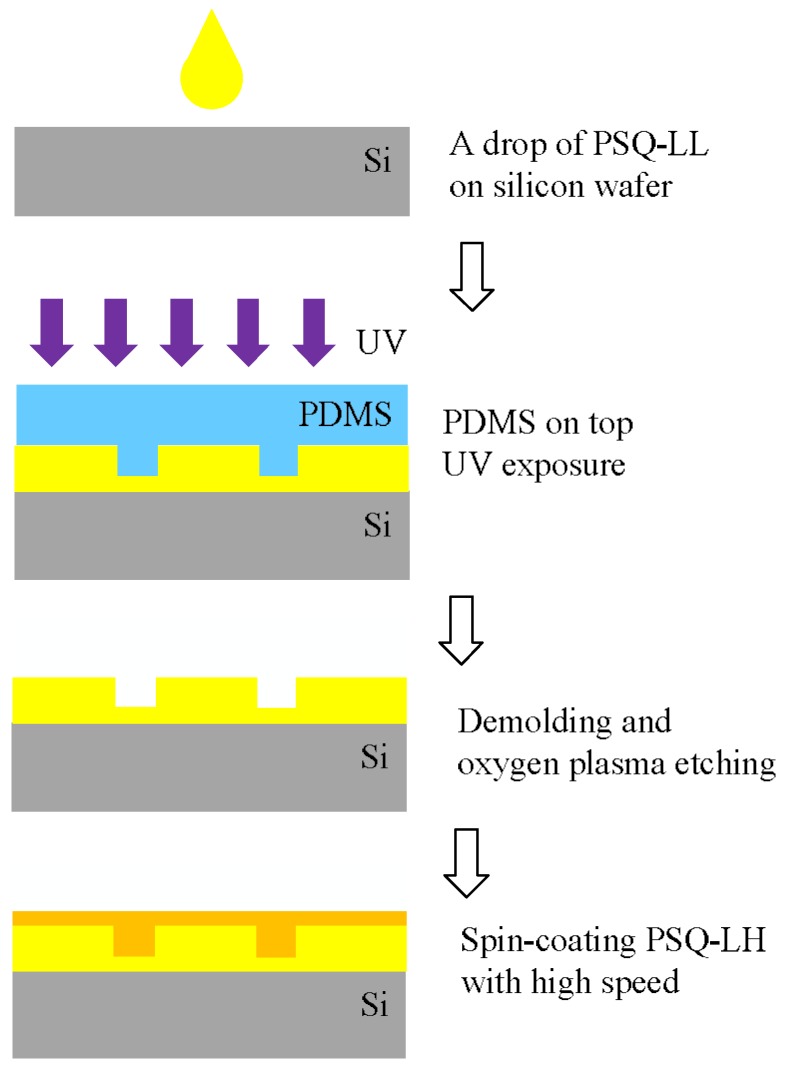
Fabrication process of the inverted ridge waveguide using UV soft imprinting technique [35].

In [35], the microring resonator with inverted ridge waveguide using the UV soft imprinting technique is presented. The polymer polysiloxane-liquid (PSQ-L) with two forms of a high index polymer PSQ-LH and a low index polymer PSQ-LL are utilized as the core and cladding materials for the inverted ridge waveguide. PSQ-L is a kind of silicate-based inorganic–organic hybrid polymer with properties of purely liquid (solvent free) and UV curable, which is compatible with soft-lithography [45]. Figure 3 shows the fabrication process of the inverted ridge waveguides. The cladding layer rather than the core layer is imprinted to form the groove, and then the core waveguide is defined using spin-coating step to fill the groove. This process smartly avoids the difficulty of controlling the thickness of residual layer in the conventional imprinting process with ridge structure. The thickness of the slab layer can be made thin enough by increasing the spin-coating speed or using diluted core polymer material. In addition, the structure imprinted in the cladding layer is helpful for forming the core waveguide using other techniques, such as ink-jet imprinting [46]. 

**Figure 4:**
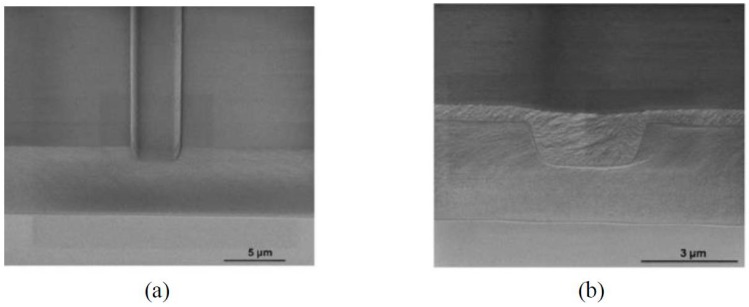
The SEM pictures of (**a**) the imprinted low index layer and (**b**) the waveguide cross section after spin-coating the high index layer [35].

**Figure 5:**
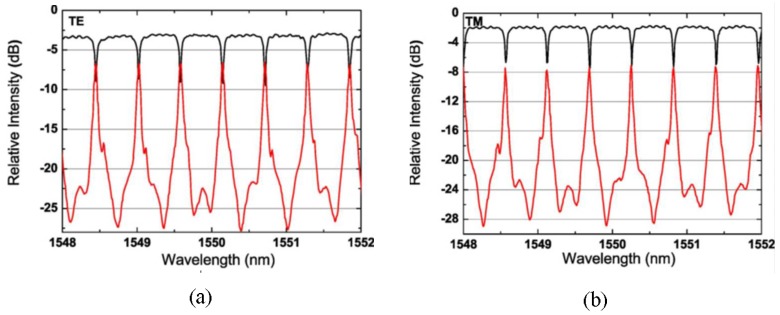
Measured transmission spectra of the inverted ridge waveguide microring resonator operating at (**a**) TE polarization and (**b**) TM polarization [35].

Figure 4a,b shows the scanning electron microscope (SEM) pictures of the imprinted low index layer and the waveguide cross section after spin-coating the high index core layer. Due to the high spin-coating speed, the core layer forms a shallow concave on the waveguide surface. It has no influence on the performance of the device, since the most light is confined in the ridge of the core layer. The racetrack microring resonator based on the inverted ridge waveguide is fabricated using the UV soft imprinting technique, with a radius of 400 μm, a gap between parallel straight waveguides of the coupler of 1.2 μm, and a coupling length of 150 μm. The transmission spectra of the device are measured using coupling light from a tunable laser to the waveguide operating at TE polarization and TM polarization, respectively. The measurement results are shown in Figure 5. Since the TM mode shows a similar response to the TE mode, the following analysis is, therefore, done for TE polarization only. The free spectrum range (FSR) of the microring is about 0.57 nm. The full-width at half-maximum (FWHM) is about 0.037 nm. By taking the ratio between the resonance wavelength and FWHM, a quality (Q) factor of about 4.2 × 10^4^ is calculated. It is expected that the polarization-independent waveguides can be fabricated by optimizing the waveguides dimension.

#### 3.1.2. Microring Resonators with Ridge Waveguide

Photonic waveguide devices with ridge structure are more desirable for some applications, especially for biosensors. There will be much interaction between the optical evanescent wave and the tested biological sample, resulting in a high sensitivity. However, the residual slab layer of the imprinted ridge waveguide is usually very thick, which limits the minimum radius of microring, as well as the FSR. Some researchers have expended a lot of effort to solve it, but usually the proposed solutions require expensive imprinting equipment or a specific setup, for example, the vacuum-assisted microfluidic technique [47]. The selective imprinting technique was developed in our group to reduce the thickness of the residual slab layer [36]. 

**Figure 6:**
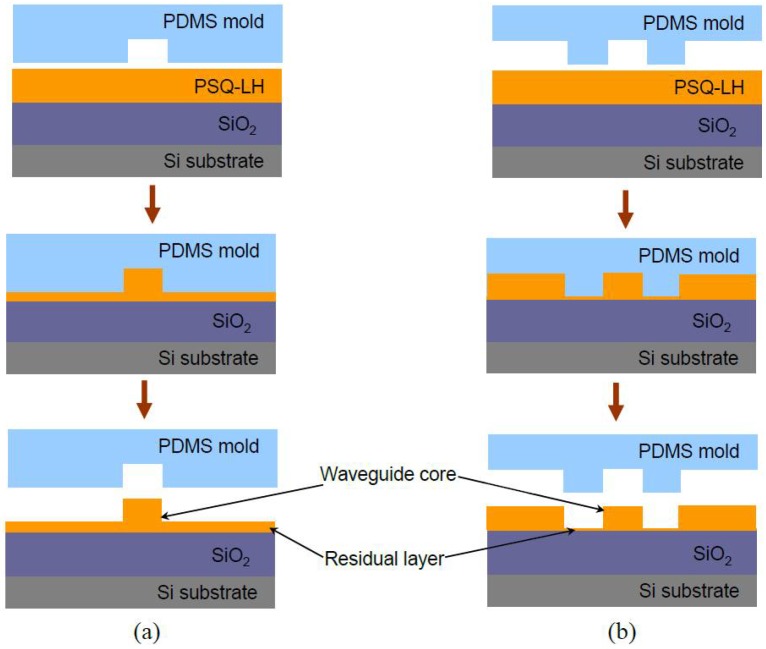
The schematic process of (**a**) the conventional imprinting technique and (**b**) the selective imprinting technique [36].

**Figure 7:**
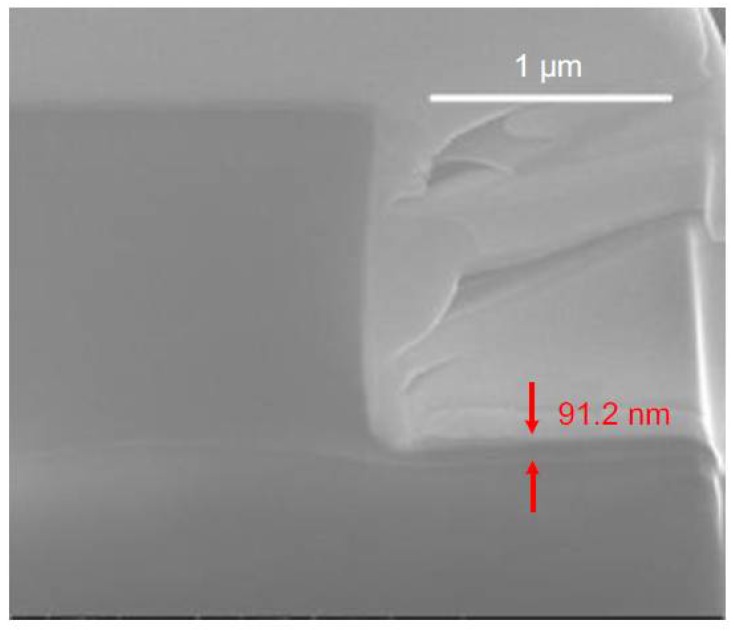
The SEM picture of the fabricated ridge shaped waveguide with negligible residual layer thickness [36].

The schematic processes of the conventional imprinting technique and the selective imprinting technique are illustrated in Figure 6. Instead of using the single trench pattern mold to directly imprint the waveguide into the core layer as shown in Figure 6a, the ridge shape waveguide is formed by imprinting two trenches on both sides of the waveguide (see Figure 6b). Only a small amount of the polymer needs to be squeezed by the mold. Therefore, the resistance of the polymer against the mold is low, and the requirement for the strength of the force exerted on the mold can be minimized greatly. During the selective imprinting process, the polymer waveguide is patterned only by the capillary forces between the PDMS soft mold and the substrate, without any additional imprinting tools. A polymer waveguide with good rectangular shape, as well as negligible residual slab layer thickness (below 100 nm), fabricated with this technique is shown in Figure 7.

**Figure 8:**
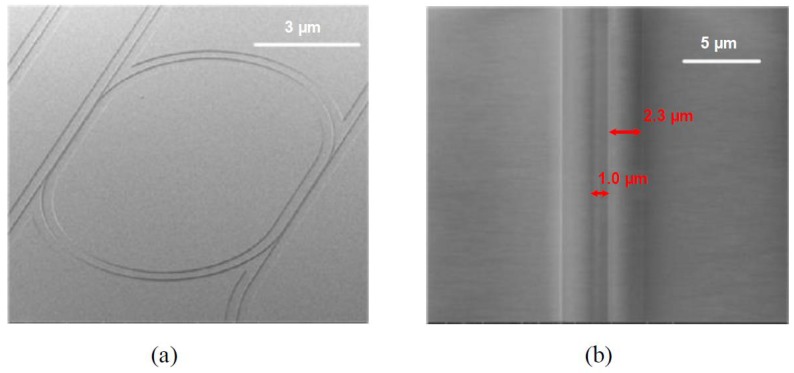
The SEM pictures of the racetrack microring resonator fabricated by the selective imprinting technique. (**a**) Image of the entire structure; (**b**) detail of the coupling region [36].

**Figure 9:**
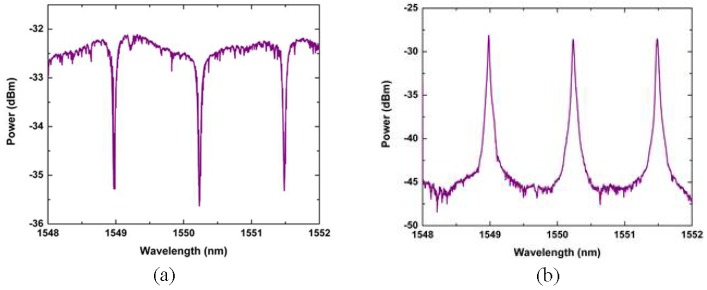
Measurement results of the polymer microring resonator with ridge waveguide. (**a**) Through port; (**b**) drop port.

The ridge waveguide racetrack microring resonator with negligible residual layer is fabricated using the selective imprinting technique. Figure 8 shows the SEM images of the entire structure and the detail of the coupling region. Because all the consisted waveguides are with air cladding, a small transformation of the ring structure into a racetrack is made as well in order to increase the coupling. Figure 9 gives the measurement results for the racetrack microring resonator with a radius of 150 µm and Q-factor as high as 5 × 10^4^. The FSR is increased to 1.252 nm compared with the inverted ridge waveguide-based microring, while no degradation of the performances is observed. From this result, it can be concluded that no significant bending loss can arise from the residual layer, which has been reduced to a very thin level during the imprinting process.

### 3.2. Optical Waveguide Splitters

Optical splitters, also known as splitters, are one of the most important passive devices in optical fiber communication system. With the rapid development of telecommunication system, especially the fiber-to-the-home (FTTH), the need for high-performance, low-cost splitters has become increasingly urgent. The fiber-based optical splitters with one input and multiple outputs are usually utilized. However, the output uniform of the divided power and the insertion loss will deteriorate when the number of outputs increases. The photonic integration technique provides a potential way with which to fabricate optical waveguide splitters with small size and good performance [48,49]. With the help of imprinting technique, polymer-based optical waveguide splitters can be fabricated with low cost and low insertion loss. 

**Figure 10:**
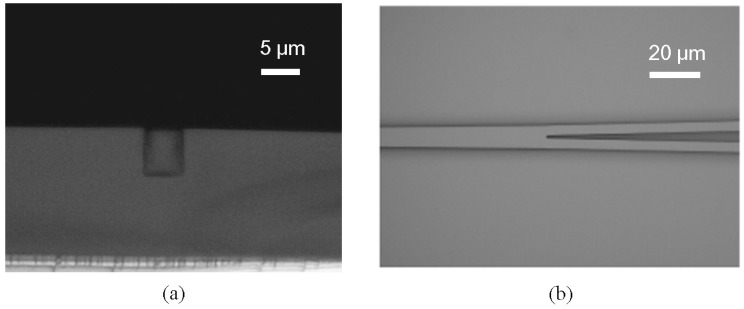
Cross-section view showing rectangular core shape and no residual layer on the waveguide top; (**b**) the optical microscopic image of Y-junction area [50].

Y-branch-based optical waveguide splitter with preliminary demonstration of 1 × 4 structure is fabricated using the UV soft imprinting technique [50]. Polymer materials used in the experiment are fluorinated acrylate resin, LFR (ChemOptics, Daejeon, Korea), with the refractive indices ranging from 1.375 to 1.395 at 1550 nm wavelength. PDMS mold is replicated from a silicon mold through a casting molding method and properly cured after baking at 60 °C for 4 h. LFR with the lower refractive index is spin-coated on the silicon substrate as the under cladding layer. The grooves on the cladding layer fabricated from PDMS mold imprinting are filled with the LFR with higher refractive index as the core layer. The upper cladding layer is then spun on top using the same procedure. This inverted waveguide helps to reduce the thickness of the residual layer, which can be controlled by the spinning speed during the core layer preparation. Figure 10a shows the cross-section of the waveguide after being filled with higher refractive index LFR. It can be seen that the core shape is perfectly rectangular, and no layer is residual on the waveguide top. The optical image of Y-junction area is given in Figure 10b, which shows that the waveguide is split into two paths with a good form.

**Figure 11:**
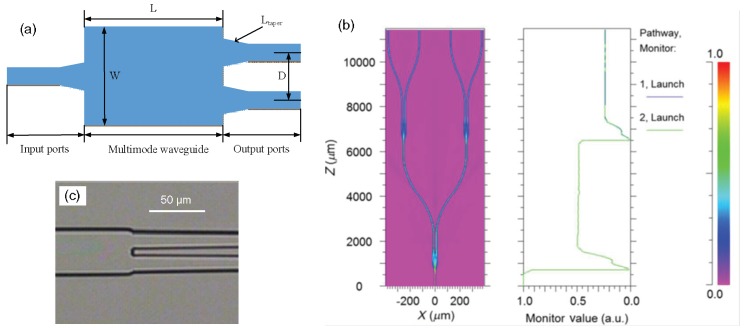
(**a**) Schematic of the MMI-based waveguide splitter; (**b**) the simulated beam propagation in 1 × 4 waveguide splitter; and (**c**) the microscopic image of the connection section between the multimode waveguide and the two single mode waveguides [52].

During the imprinting process, the deformation of the waveguide structure and the Y-junction area is crucial for the splitter performance [51]. It is found that sometimes the Y-junction is demolded with defects after demolding due to the ultra-narrow gap at the splitting section. In order to solve this problem, the multimode interference (MMI)-based optical waveguide splitter is designed and fabricated using the similar UV soft imprinting technique [52]. The MMI based splitter, shown in Figure 11a, which avoids the narrow structure as Y-junction based splitter, is tolerant of the structure deviations by the fabrication process. A taper is introduced between the multimode waveguide and single mode waveguide for lower coupling loss. According to the self-imaging theory of multimode waveguide, the MMI-based waveguide splitter is optimized with the structure parameters *L* = 850 µm, *D* = 22 µm, *W* = 40 µm, and *L_taper_* = 350 µm. The simulated beam propagation in 1 × 4 waveguide splitter is illustrated in Figure 11b. As the splitter is expected to connect with fiber array, S-bend waveguide is applied to enlarge the distance between output ports (to be 250 μm). Figure 11c shows the microscopic image of the connection section between the multimode waveguide and the two single mode waveguides. The average insertion loss of the 1 × 4 waveguide splitter is 12.98 dB with uniformity of 1.08 dB. In addition, the average polarization-dependent loss (PDL) is measured as small as 0.05 dB due to the low birefringence of the polymer material and the square cross-section of the waveguide.

### 3.3. Arrayed Waveguide Gratings

**Figure 12:**
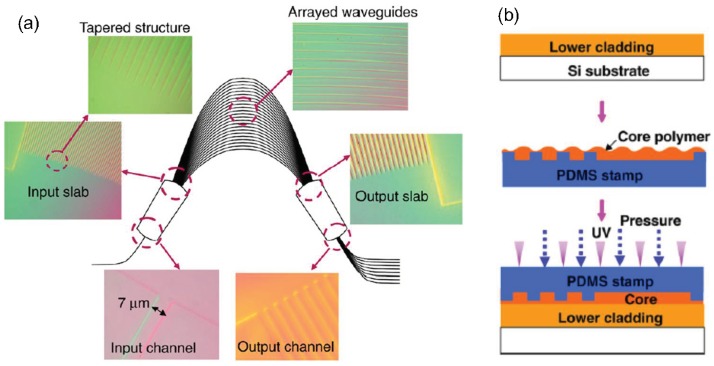
(**a**) Schematic diagram showing the plan view of the imprinted AWG device; (**b**) the reverse imprint procedure for core layer patterning [53]. Copyright 2007 Elsevier.

The arrayed waveguide grating (AWG) with the similar function of diffraction grating can be used as a wavelength multiplexer or demultiplexer. AWGs play an important role for the dense wavelength division multiplexing (DWDM) optical fiber communication system. In [53], the soft PDMS mold with AWG patterns is prepared from a fine quartz mold, which is then used to stamp on the polymer core layer with precise contact, as well as easy lift-off process. Figure 12a shows the top view of the fabricated AWG, in which ZPU12-450 with refractive index of 1.45 at wavelength of 1.55 μm and ZPU12-430 with refractive index of 1.43 at wavelength of 1.55 μm from ChemOptics are utilized as core and cladding, respectively. It can be seen from Figure 12a that there are mainly five parts in the AWG, including a single input waveguide, two slab waveguide, a group of arrayed waveguides, and multiple output waveguides. Except the slab waveguide, the waveguide is basically a single mode one. In order to improve the lightwave coupling efficiency between the slab waveguide and the single mode waveguide, a tapered structure has been incorporated in between. A reverse imprint procedure is applied, as shown in Figure 12b, incorporating a PDMS stamp, in which the waveguide patterns are formed in the core instead of the lower cladding without resorting to spin-coating process. In this way, the height of the pattern can be determined using the precisely prepared PDMS stamp. In addition, the thickness of the residual layer can also be controlled by pressing PDMS against the lower cladding with various strengths, as well as varying the viscosity and amount of the core polymer. An 8-channel AWG is fabricated showing a 0.8 nm spacing with the center wavelength ranging from 1543.7 to 1548.3 nm. The 3 dB bandwidth is about 0.8 nm, and the channel crosstalk is approximately 10 dB. It is found that the existence of the undesired residual layer caused bandwidth broadening, which is attributed to the mismatch between the output channel and the focal length of the beam. This unwanted residual layer may be improved using sol-gel polymer materials [54] or by introducing further post-etching processes. 

### 3.4. Long-Period Waveguide Gratings

**Figure 13:**
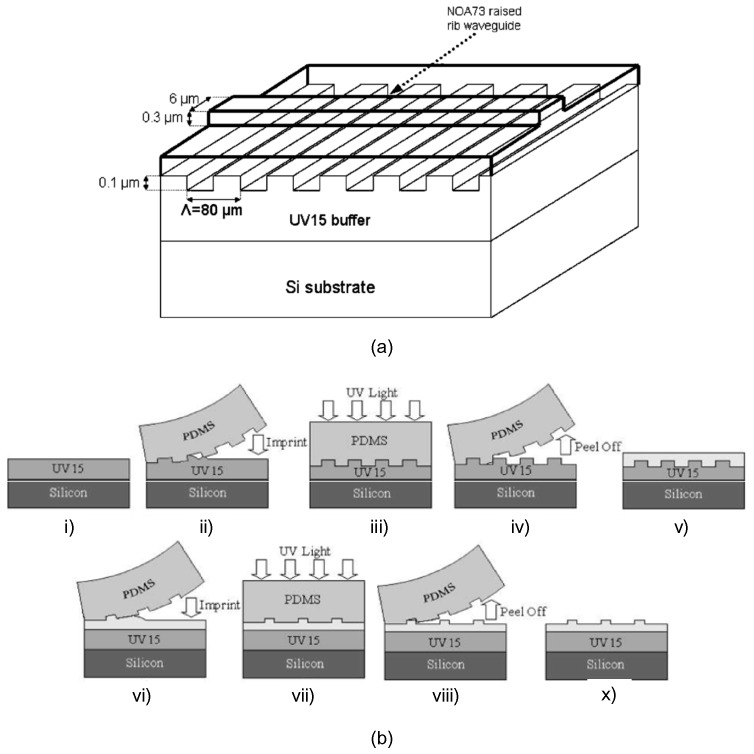
(**a**) Schematic structure of the LPWG; (**b**) two processes for imprinting the grating waveguides (i)–(v) and for rib waveguide (vi)–(x) [56]. Copyright 2005 IEEE.

Long-period gratings have various applications, such as sensors, band-rejection filters, and gain flatteners for erbium-doped fiber amplifier (EDFA). During the manufacturing process, the conventional long-period fiber gratings are limited by the geometrical size of the fiber and materials. To overcome this issue, long-period waveguide gratings (LPWGs) have been demonstrated with large variety of materials and different fabrication methods to select from [55]. In [56], the UV soft imprinting technique is utilized to fabricate a LPWG. Figure 13a shows the schematic structure of the LPWG, in which the rib waveguide is raised by the periodic grating on the cladding-core interface. There are two processes for fabricating the LPWG with UV soft imprinting technique, as illustrated in Figure 13b. The first process is to imprint the grating waveguides as the steps shown from (i) to (v) in Figure 13b. The cladding layer is using polymer material UV15. A PDMS soft mold with 80 μm period grating patterns is used to stamp on the uncured UV15 layer due to its good flexibility for its better accuracy for structure replication. The second process is to imprint the rib waveguide as the steps shown from (vi) to (x) in Figure 13b. Another PDMS mold with waveguide feature is then applied onto the top cladding layer to form the rib waveguide. Polymer waveguides are diced with a high-speed polishing saw, which can form nice end-faces for butt-coupling tests afterwards. It is found that the resonance of the grating occurs at 1585 nm with a 3 dB bandwidth of 12 nm and rejection level of 10 dB. These results show that the UV soft imprinting technique has the capability to fabricate three-dimensional functional photonic devices [36].

### 3.5. Microlenses

**Figure 14:**
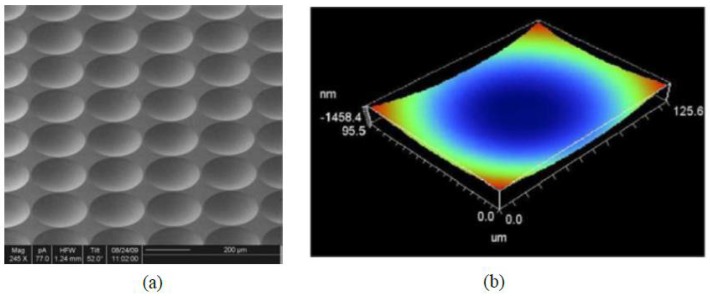
(**a**) SEM image of the replicated microlenses into the resist PAK-01; (**b**) surface profile of an imprinted microlens [42]. Copyright 2010 Elsevier.

Microlenses and microlens arrays (MLAs) have shown promising ability for light collection and collimation, with a large range of applications including imaging sensors, interconnects, and photodetectors. Various fabrication methods for MLAs have been investigated. However, the fabrication cost is still too high for large-scale production due to the complex steps involved [57]. In [42], the polymer 40 × 40 microlens array with a pitch of 250 μm, a diameter of 240 μm, and a sag height of 24.5 μm have been prepared. Microlenses are replicated into the UV curing polymer material PAK-01 by step and stamp UV imprinting technique on silicon substrates with a diameter of 150 mm. The resulting substrates are used as masters to cast PDMS templates. With the optimized dispense strategy, including the adjustment of the dispense height, pressure, and amplitude, air bubbles can be avoided in the dispensed resist layer. Figure 14a shows the SEM image of the replicated microlenses into the resist PAK-01. The surface of imprinted microlens profile measured by a white light profilometer is shown in Figure 14b.

## 4. Active Photonic Integrated Waveguide Devices

Compared with the inorganic optical materials, such as silica, glass, and SiN_4_, organic polymers have large TO coefficient, which has the potential to decrease the power consumption of TO integrated waveguide devices [58,59]. This section gives some imprinted active polymer waveguide devices based on TO tuning effect.

### 4.1. Tunable Microring Resonator Filters

**Figure 15:**
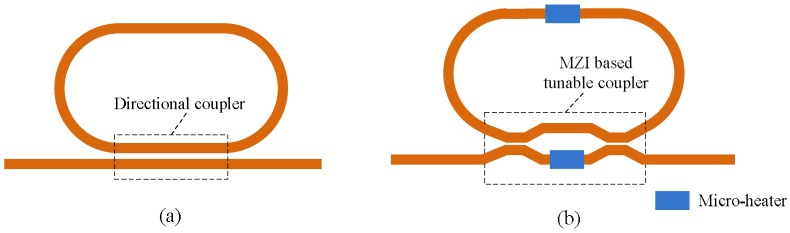
Schematic structures of the microring resonators consisting of (**a**) a directional coupler and (**b**) a MZI based tunable coupler.

As mentioned in Section 3.1, the microring resonators are the key unit in the photonic integrated circuit. It is desired that the coupling coefficient or the resonant wavelength of the microring resonator can be tuned according to the requirement of applications [60,61]. Figure 15b shows the schematic structure of the tunable microring resonator, in which the Mach-Zehnder interferometer (MZI) with a micro-heater on one of its arms is introduced to replace the conventional directional coupler, as shown in Figure 15a, while another micro-heater is placed on the microring waveguide. Consequentially, the coupling coefficient and the resonant waveguide can be tuned with micro-heaters.

**Figure 16:**
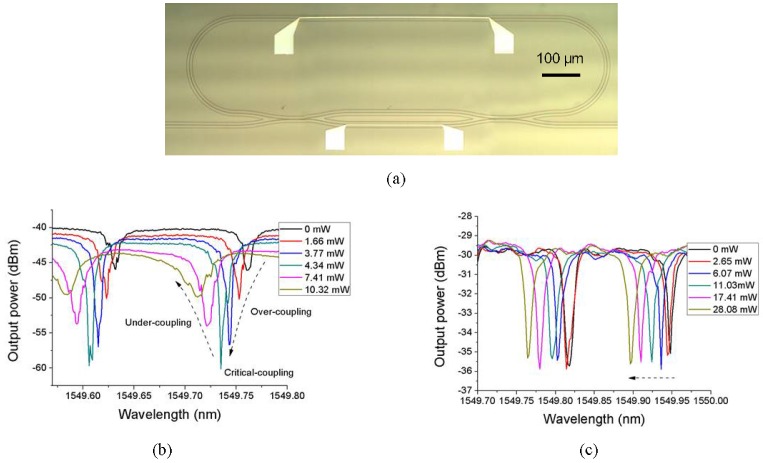
(**a**) The optical microscopic image of the fabricated polymer waveguide filter; (**b**) filter response spectra of the microring resonator by tuning the MZI based coupler; and (**c**) resonant wavelength tuning response [62].

The MZI-based polymer microring resonator is fabricated using the UV soft imprinting technique, and then the micro-heaters are patterned using the lift-off process [62]. The optical microscopic image of the tunable microring resonator device is shown in Figure 16a. The tunable properties of the fabricated polymer microring resonator are characterized. Different coupling states [60], including “over-coupling”, “critical coupling”, and “under-coupling”, are realized by applying the power only to the micro-heater on the MZI arm, as shown in Figure 16b. The controllable equivalent coupling coefficient is very useful for the application of microring resonator-based tunable time delay lines [63]. It is noted that such equivalent coupling coefficient tuning during this process generates an additional phase shift to the microring resonator, which results in a small resonant wavelength shift. It can be compensated for by tuning the phase shift on the microring waveguide. The resonant wavelength of the fabricated polymer microring resonator can also be tuned. This is realized by applying the power to the micro-heater on the microring waveguide, as the measured results show in Figure 16c. It can be seen that the resonant wavelength may be tuned freely within the FSR around 0.13 nm.

**Figure 17:**
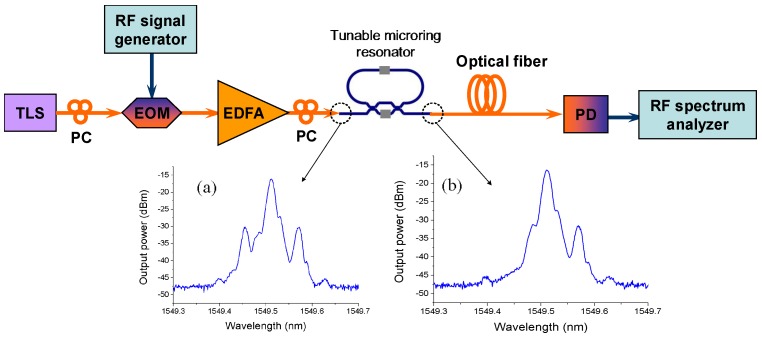
Experiment setup for RF signal transmission over fiber by using the tunable microring resonator as a notch filter, in which the insets (**a**) and (**b**) show the measured optical spectra of RF modulated lightwave before and after the microring resonator [62].

The fabricated tunable microring resonator is utilized as a notch filter to suppress one sideband of the radio frequency (RF) modulated lightwave signal with the output of single sideband (SSB) signal. The SSB signal can overcome the chromatic dispersion-induced power fading effect for high frequency RF signal transmission over a long fiber [64]. The experimental setup is shown in Figure 17, in which the insets (a) and (b) show the measured optical spectra of RF (7.2 GHz) modulated lightwave before and after the microring resonator. It can be seen that about 18 dB of the left sideband is suppressed. To demonstrate the validity of SSB filtering response of the microring resonator, Quadrature Phase Shift Keying (QPSK) signal of 1 Msps carried by 7.2 GHz microwave is transmitted over 70 km single mode fiber (SMF). For comparison, the double sideband (DSB) signal transmission is also tested, in which the equal insertion loss of the integrated waveguide chip is provided by a tunable optical attenuator. After 70 km SMF transmission, the error vector magnitude (EVM) of SSB carrying signal is 22.4%, while the one of DDB carrying signal is 61.3%, which proves the feasibility of the microring resonator as a notch filter.

### 4.2. Tunable Waveguide Bragg Grating Filters

**Figure 18:**
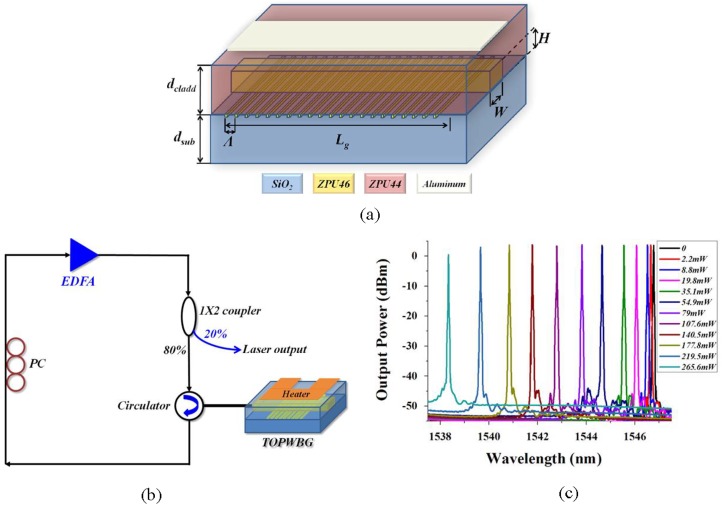
(**a**) The structure of the TOPWG; (**b**) the schematic of the proposed tunable fiber ring laser with TOPWBG as the wavelength selective filter; and (**c**) the output spectra of the tunable fiber ring laser with different heating power [67]. Copyright 2015 Elsevier.

Optical waveguide Bragg grating, as a basic waveguide filtering element, can be utilized to select a certain wavelength for external cavity lasers [65] or the channel selective receiver in the optical fiber network [66]. A tunable TO polymer waveguide Bragg grating (TOPWBG), with a structure as shown in Figure 18a, is fabricated using the imprinting technique [67]. The TOPWBG is measured with a reflectivity of 25 and 3 dB bandwidth of 0.8 nm. It is used as a wavelength selective filter in the ring laser system, as shown in Figure 18b. The established tunable laser exhibits an output of 4.1 dBm, side-mode suppression ratio of 55, and 3 dB bandwidth of less than 16 pm. By driving the heater with different powers, the laser wavelength can be tuned due to the TO tuning of the Bragg wavelength of the TOPWBG. The measured laser output spectra with different heating powers are shown in Figure 18c with the wavelength tuning range of 8.4 nm. According to the relationship between the heating power and the laser wavelength, a good linearity of about 32 pm/mW is obtained.

### 4.3. Optical Switches

**Figure 19:**
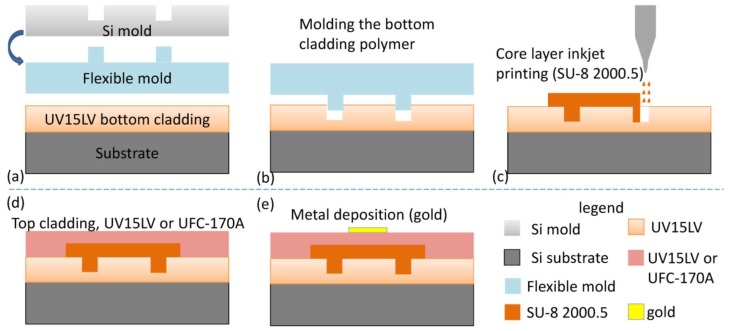
Process flow for fabricating a 2 × 2 thermo-optic polymer switch using imprinting and ink-jet printing method. (**a**) Silicon and flexible mold; (**b**) imprinting the bottom cladding; (**c**) core layer deposition; (**d**) top cladding deposition; and (**e**) metal electrode deposition [46]. Copyright 2013 OSA.

Optical switches and related arrays play important roles in many applications, such as optical routing [68], optical add–drop multiplexing [69], and optical true time delay [70] in the DWDM optical fiber communication system and the optical signal processing system. A printable TO switch is demonstrated by utilizing imprinting and ink-jet printing techniques [46]. A polymer material named UV15LV with refractive index of 1.501 at wavelength of 1.55 μm from MasterBond is selected as bottom cladding layer, SU8-2000.5 with refractive index of 1.575 at wavelength of 1.55 μm from MicroChem as core layer, and UFC-170A with refractive index of 1.496 at wavelength of 1.55 μm from URAY Co. Ltd. (Austin, TX, USA) as the top cladding layer. Figure 19 illustrates the process flow for fabricating a 2 × 2 TO polymer total-internal-reflection (TIR) switch. The UV imprinting process is utilized for transferring the pattern from the soft mold to the bottom cladding polymer. Then, the core layer is ink-jet printed on top; following this step, another layer (top cladding layer) is ink-jet printed on the top of the core layer. The last step involves depositing a gold heating electrode on the top. 

**Figure 20:**
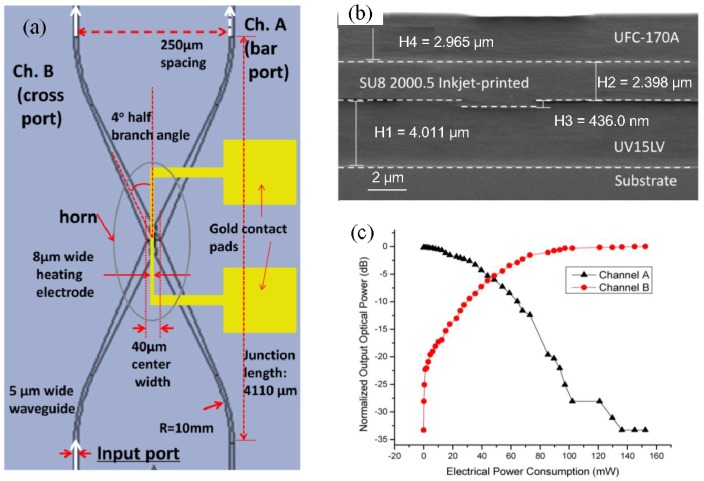
(**a**) Schematic showing the top view of the 2 × 2 TO TIR switch layout; (**b**) SEM cross-section view of printed layers in the TO polymer switch; (**c**) normalized optical output power versus electrical power from both bar port (Channel A) and cross port (Channel B) [46]. Copyright 2013 OSA.

Figure 20a shows the schematic of the 2 × 2 TO polymer switch with a horn structure [71] at the junction to construct the TIR structure. The parameters of the TIR switch are optimized for cross-talk minimization and low switching power. The X junction is 4°, and the width of the heating element at the center is 8 μm. The SEM cross section image of the fully printed device is shown in Figure 20b. The performance of fabricated TO polymer switch at different applied voltages is characterized. The relationship between the normalized optical output power from two output channels and the electrical power consumption is shown in Figure 20c. It can be seen that the power for the cross port to reach its maximum output is about 100 mW, and the cross-talk is over 32 dB. The response speed of the fabricated TIR switch is measured with a rising/falling time of less than 0.5 ms and an operation frequency of up to 1 kHz. For the true time delay (TTD) application, utilizing large-area imprinting (with large-area flexible PDMS mold) and ink-jet printing process, a 4-bit TO switch-based TTD module has been successfully fabricated [41], which is capable of providing sufficient time delay for achieving ±60° steering angle in an X-band phased array antenna system.

### 4.4. Variable Optical Attenuators

**Figure 21:**
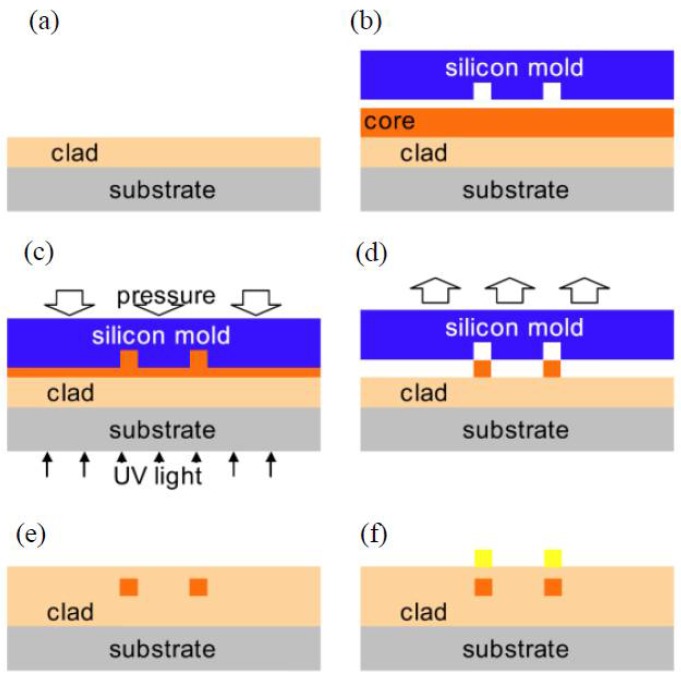
UV imprinting steps for integrated waveguide VOA-based on polymer materials. (**a**) Spin-coating and curing for under-cladding; (**b**) core material dispensing; (**c**) imprinting under pressure with UV irradiation; (**d**) detachment of the mold; (**e**) spin-coating and curing for cover-cladding; and (**f**) TO electrode patterning [74]. Copyright 2006 Elsevier.

Variable optical attenuators (VOAs), which enable dynamically flexible optical power regulation, have a lot of applications in the photonic integrated circuits and reconfigurable optical networks [72,73]. A polymer TO VOA is designed and fabricated via the UV imprinting technique in [74]. The silicon is utilized as the mold material. The quartz is applied as the substrate material due to its transparency to UV irradiation. Figure 21 illustrates the UV imprinting steps for polymer waveguide VOA.

**Figure 22:**
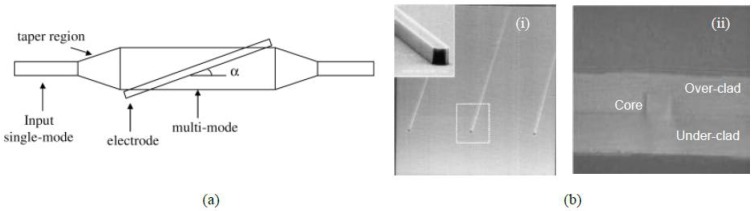
(**a**) Schematic of polymer waveguide TO VOA and (**b**) characterization of UV-imprinted single mode waveguide [74]. Copyright 2006 Elsevier.

The polymer waveguide VOA, as shown in Figure 22a, comprises a single-mode waveguide channels, taper structures, and a multi-mode waveguide channel. Figure 22b shows the fabricated waveguides: (i) SEM picture of UV-imprinted single-mode waveguide core and (ii) cross-sectional view of the fabricated polymer waveguide. The propagation loss of the straight single mode is 0.35 dB/cm at a wavelength of 1.55 μm, and the average insertion loss is less than 2 dB. The attenuation characteristics according to the applied electrical power are measured with 30 dB attenuation with electrical power of 80 mW and a rising/falling time of less than 5 ms.

## 5. Electro-Optic Modulators

Compared with inorganic materials such as the most widely used LiNbO_3_, the polymeric EO materials have many advantages including lower cost, ease of processing, and larger EO coefficients. Especially, the polymeric materials have the potential for achiving high EO coefficients by virtue of synthetization at molecular level [75,76]. For example, the long flexible chain-modified, julolidinyl-based chromophores linked by thiophene bridge and TCF acceptor (chromophore WJ5) afford the EO coefficient (r_33_) value of 266 pm/V [77]. The chromophore with thiophene-modified π-conjugation enhances the EO coefficient to be 337 pm/V [78]. 

**Figure 23:**
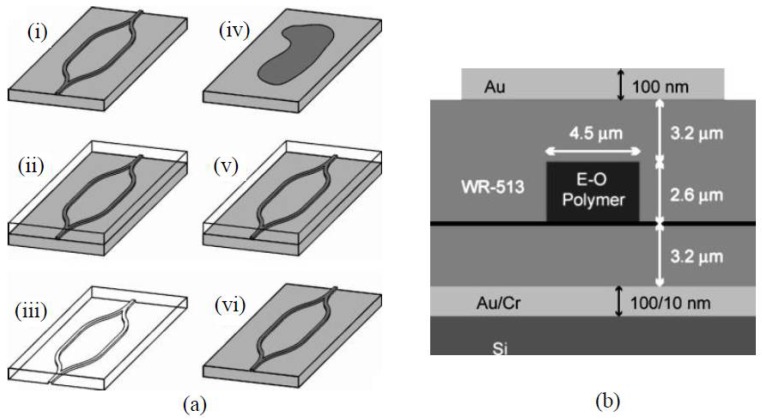
(**a**) Fabrication of the PDMS mold (i–iii) and replication of the MZI structure (iv–vi). A master MZI device (i) is patterned using photolithography of SU-8. PDMS is poured on the master device (ii), cured, peeled, and diced (iii). A drop of electro-optic core polymer solution is placed on the silicon substrate (iv). The PDMS stamp is depressed (v) until the core polymer is cured. The stamp is peeled to reveal the replicated device (vi); (**b**) cross-sectional view of the electro-optic waveguide structure with electrode [79]. Copyright 2004 AIP.

Incorporating the highly nonlinear and stable chromophore doped in amorphous polycarbonate, a MZI based EO intensity modulator is designed and fabricated using the UV imprinting technique [79]. In order to reduce the radiation loss of the Y junction, two S-shaped bends with radii of 1 mm are adopted. Figure 23a shows the process steps for the fabrication of PDMS stamp (i–iii) and the molding of EO polymer layer (iv–vi). The waveguide MZI structure is formed by the molding of the core polymer using the soft PDMS stamp. The polymer materitals with low refractive index for under cladding and upper cladding should be chosen carefully, because the solution solvent of core material must not dissolve the under cladding polymer, and the solution solvent of upper clading material must not dissolve the core polymer. The cross-sectional view of the electro-optic waveguide structure is illustrated in Figure 23b. The fabricated EO modulater is measured with the halfwave voltage (*V*_π_) of 8.4 V at 1600 nm, and the on/off extinction ratio is better than 19 dB. As the first imprinted EO polymer modulator, the halfwave voltage is relatively high. It could be improved by thinning the cladding layers in a push–pull fashion and optimizing the poling efficiency.

**Figure 24:**
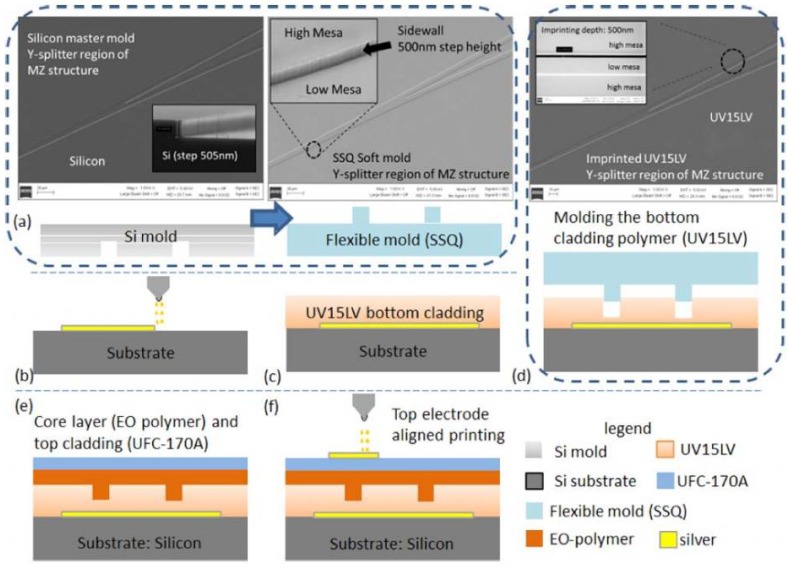
Main process flow for fabricating an EO polymer modulator using imprinting and ink-jet printing methods. (**a**) Silicon and SSQ mold; (**b**) bottom electrode deposition; (**c**) bottom cladding layer deposition; (**d**) imprinting; (**e**) core layer and top cladding layer deposition; and (**f**) top electrode deposition [80]. Copyright 2013 OSA.

Despite utilizing imprinting to pattern one layer, other material layers (metal electrode) still need to be prepared via spin-coating, evaporation, etching, or lift-off methods, which increase the process complexity and cost. Chen’s group worked toward achieving an all-printable EO modulator [80]. They demonstrated an electro-optic polymer-based MZ modulator fabricated by utilizing advanced UV imprinting and aligned ink-jet printing techniques for patterning and layer deposition. The main process flow for fabricating the MZ modulator is illustrated in Figure 24.

**Figure 25:**
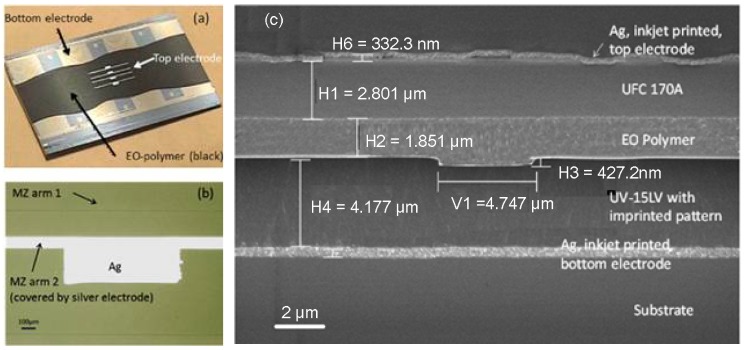
Microscope images of (**a**) printed EO polymer modulator; (**b**) ink-jet printed top electrode; and (**c**) SEM picture of the modulator cross section [80]. Copyright 2013 OSA.

The bottom electrode layer is directly patterned on the substrate by ink-jet printing. By using the UV imprinting method with a transparent flexible mold, the waveguide structure is transfered into the under cladding polymer. All other layers are ink-jet printed step by step. The top electrode is printed over the MZI arm with alignment. Besides the requirement of materials to form a optical waveguide using the imprinting technique, the core material and the electrode material should have suitable viscosity to be ink-jet printed. Figure 25a,b shows the microscope image of printed EO polymer modulator and ink-jet printed top electrode, respectively. Figure 25c gives the SEM picture of the modulator cross section. It can be seen that the imprinted trench is filled with EO polymer, and the top and ground electrodes are uniformly deposited with the top electrode located right on top of the rib waveguide.

## 6. Photonic Biosensors

Biosensors have been attracting more and more attention due to their wide applications in the fields of clinical diagnosis, environmental monitoring, new drug development, and food safety. Photonic biosensors offer distinct advantages for sensing applications compared with their electronic counterparts, including electrical passiveness and insensitivity to electromagnetic interference. For the requirements of label-free and real-time monitoring of the dynamics of biomolecular reactions, the photonic integrated waveguide biosensors can avoid the labor and time consuming process of the conventional methods, such as the enzyme-linked immunosorbent assay (ELISA). Furthermore, these planar waveguide photonic biosensors could be integrated with other electronic, optical, and microfluidic components, which is helpful for the realization of lab-on-chip devices [81]. Among different materials, polymers have been proven to be ideal material platforms for photonic integrated waveguide biosensors. Besides the advantages of low cost, tunable properties, and easy fabrication, compared with inorganic materials, biocompatibility is an especially important and unique feature of polymers. Accordingly, the biochemical surface treatment of the polymer waveguide devices can be greatly simplified. This section presents the recent work about polymer waveguide photonic biosensors using the UV imprinting technique.

### 6.1. Microring Based Biosensors

Among the various structures for integrated waveguide photonic biosensors, waveguide microrings are key components due to their high Q-factor, compactness, and potential high-density array for sensing multiple targets [82,83,84]. The high Q-factor enables the lightwave to travel round in the waveguide ring, resulting in the enhanced interaction between the optical evanescent field and the analyte. Compared with other sensor structures [85], it can provide a higher sensitivity with the same area. The polymer microring-based biosensor with a ridge shape waveguide structure is fabricated using the selective imprinting technique [36]. The fabricated polymer microring is a racetrack with a radius of 350 μm. The gap and coupling length between the straight waveguide and the microring waveguide are 1 and 70 μm, respectively. 

**Figure 26:**
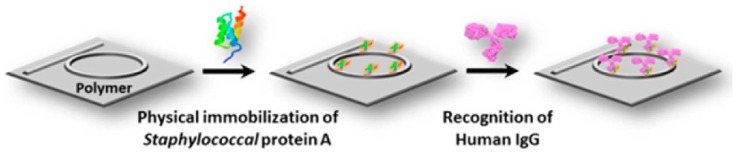
Schematic of the biosensing process using PA/IgG affinity couple [9].

**Figure 27:**
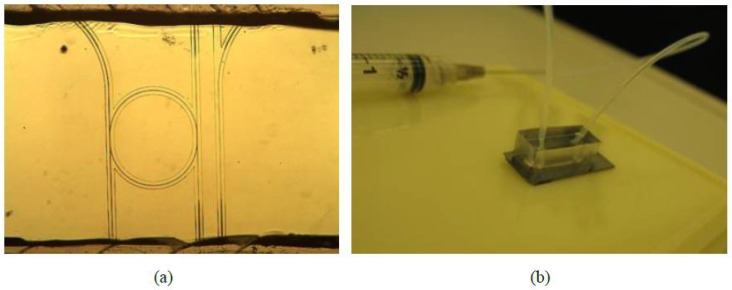
(**a**) The fluidic channel aligned with the ring resonator on the chip and (**b**) a biosensor assembled with fluidic channel and connected to a syringe.

Human-IgG (Human Immunoglobulin G) and PA (Staphylococcal protein A) are chosen as an affinity model for specific antigen-antibody sensing, as shown schematically in Figure 26 [9]. The physical immobilization via direct incubation is applied for surface functionalization, which is much simpler for polymer-based optical chips than the covalent immobilization methods. With the “stamp-and-stick” method [86], the PDMS fluidics channel is mounted on top of the microring resonator chip, as shown in Figure 27a. The biosensor assembled with fluidic channel and connected to a syringe is shown in Figure 27b.

**Figure 28:**
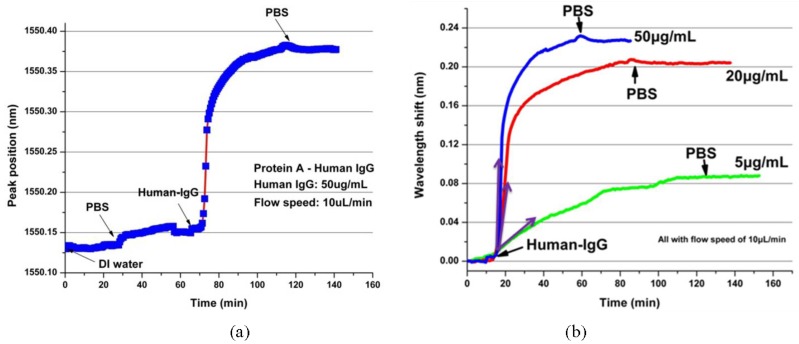
(**a**) The monitoring process of the polymer-based microring biosensor for the surface binding sensing and (**b**) the responsivities of the photonic biosensor with different concentrations of Human-IgG [36].

Surface binding sensing is measured with the following steps. With a flow speed of 10 μL/min, deionized (DI) water, and 0.01× phosphate buffer solution (PBS), Human-IgG with a concentration of 50 μg/mL and 0.01× PBS are pumped into the fluidics channel consecutively. During the process, the output resonant peak position is measured and recorded as a function of the time, with the results shown in Figure 28a. It can be seen from Figure 28a that a wavelength change of about 230 pm is caused by the affinity of Human-IgG for the protein A molecules immobilized on the chip surface as bio-receptors. The strong binding strength between the protein A and Human-IgG and the coating of protein A to the chip surface is confirmed, because no obvious reverse wavelength shift is observed in the last phase of PBS rinsing. Then, the biosensors operating at different concentrations of Human-IgG solutions are investigated, with the results shown in Figure 28b. It indicates that the photonic biosensor exhibits different dynamic responsivities to the antibody concentrations, with a detected concentration of Human-IgG as low as 5 μg/mL. 

**Figure 29:**
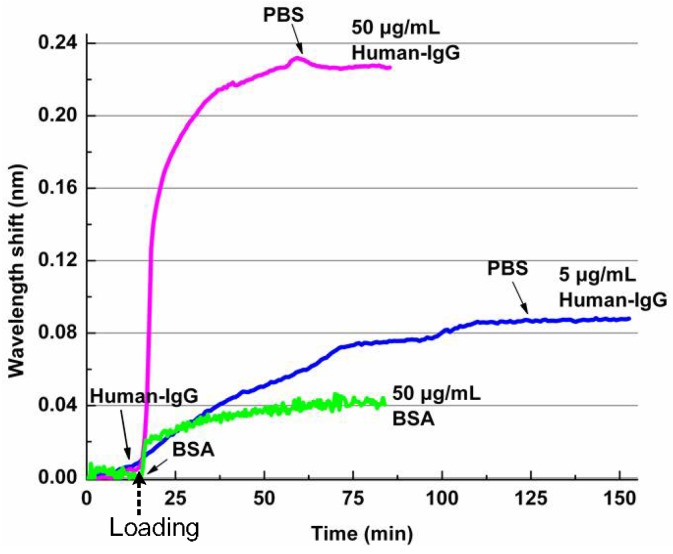
The responsivities of the PA surface functionalized photonic biosensor to specific and nonspecific interactions. Resonant wavelength shift induced by the interaction with human IgG (50 and 5 μg/mL) and with BSA (50 μg/mL) [9].

The nonspecific binding is another important aspect of label-free photonic biosensors. To test the response of polymer-based photonic biosensor toward non-targeted biomolecules, Bovine Serum Albumin (BSA) is chosen as a control protein and brought into contact with the PA-coated microring surface [9]. The concentration of the BSA for the test is 50 μg/mL (in 0.01 Mol PBS, pH 7.4). From the measured results as shown in Figure 29, it can be seen that a resonant wavelength shift of about 40 pm is caused by the nonspecific binding of the BSA molecules, and the saturation is obtained after 40 min with the BSA solution injection. As a contrast, a resonant wavelength shift of 90 pm using the specific binding of the Human-IgG with a concentration of 5 μg/mL is observed. Therefore, the recognition ability of the PA-coated microring biosensors is validated.

### 6.2. Young’s Interferometer Based Biosensors

**Figure 30:**
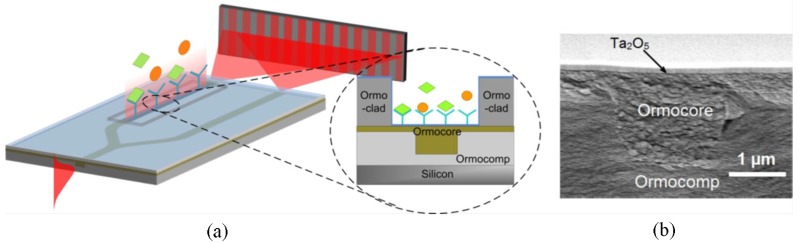
(**a**) Schematic image of the integrated YI biosensor based on inverted-ridge waveguide; (**b**) cross-section SEM image of the inverted ridge waveguide [88]. Copyright 2012 OSA.

Interferometers, such as MZIs and Young’s interferometers (YIs), are considered as one of the most sensitive devices [87] for optical sensing due to the background compensation between the sensing and reference arm against environmental disturbances, such as temperature fluctuations. In [88], a YI-based biosensor utilizing layered polymeric-inorganic composite waveguide configuration is developed. The schematic structure of the YI-based photonic biosensor is illustrated in Figure 30a. The lightwave is split into a reference arm and a sensing arm via the Y shaped splitter. Instead of recombining the two arms as in the MZI [85], the optical output from the two arms transmits to a detector screen to form interference fringes. The shift in the interference fringes is induced by the phase change on the sensing arm, which can be utilized to monitor the concentration of the analytes.

The materials used for sensor fabrication consist of the UV-curable Ormocer series of hybrid polymers (Micro resist technology GmbH). Ormocomp, Ormocore, and Ormoclad, having refractive indexes of 1.520, 1.553, and 1.536 at wavelength of 633 nm, are used for the under cladding, core, and upper cladding, respectively. The polymer-inverted ridge waveguide is fabricated using the imprinting technique with two main steps: patterning the waveguide under-cladding and filling the patterned grooves with the core material [89]. A thin layer of Ta_2_O_5_ with a high index (*n* = 2.1) is deposited on top of polymer layers, as shown in Figure 30b, which pushes the guided mode field up towards the surface, resulting in the enhanced interaction between the optical field and biomolecules under test [90].

**Figure 31:**
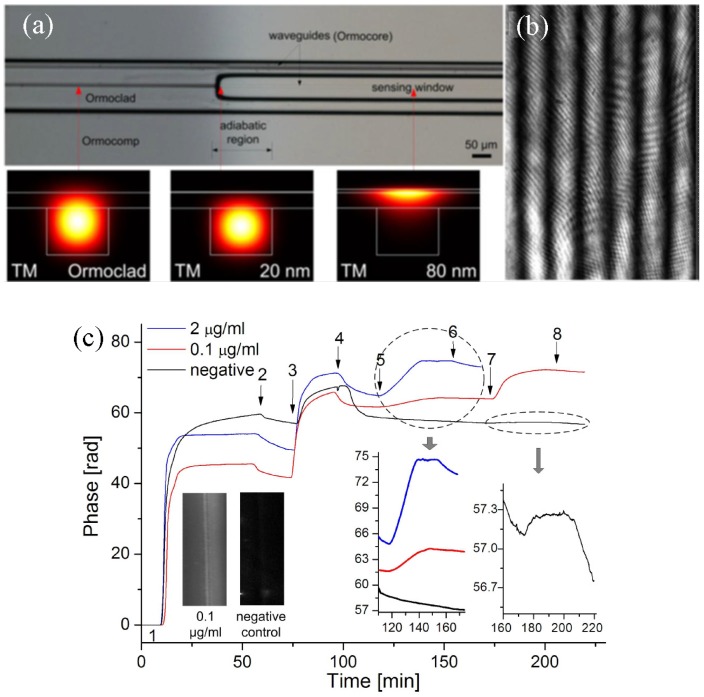
(**a**) Microscope image of the fabricated YI biosensor with a high-index Ta_2_O_5_ coating; (**b**) the generated interference pattern on the detector screen at a distance of 2.5 mm; and (**c**) the measured phase responses of the molecular binding events between surface attached CRP antibodies and different concentrations of CRP antigens [88]. Copyright 2012 OSA.

Figure 31a shows the top view microscope image of fabricated, integrated YI with a distance between the sensing and referencing waveguide of 50 μm, in which the simulated intensity profiles of the propagating TM modes are also displayed. It can be seen that by coating Ta_2_O_5_ on top of polymer layer, the guided mode field is pushed up towards the surface. The generated fringe pattern from the referencing waveguide and sensing waveguide is shown in Figure 31b. By using two-dimensional fast Fourier transform, the fringe pattern phase shifts can be analyzed with good resolution by virtue of the clear visibility of the interferogram. The specific molecular binding between C-reactive protein (CRP antigens) and monoclonal anti-human C-reactive protein (CRP antibodies) is chosen to characterize the performance of the developed YI photonic biosensor. The monitored phase responses are plotted in Figure 31c. Considering the noise level of 0.003 rad, the detection limit for surface sensing can be estimated to be about 100 fg/mm^2^. 

**Figure 32:**
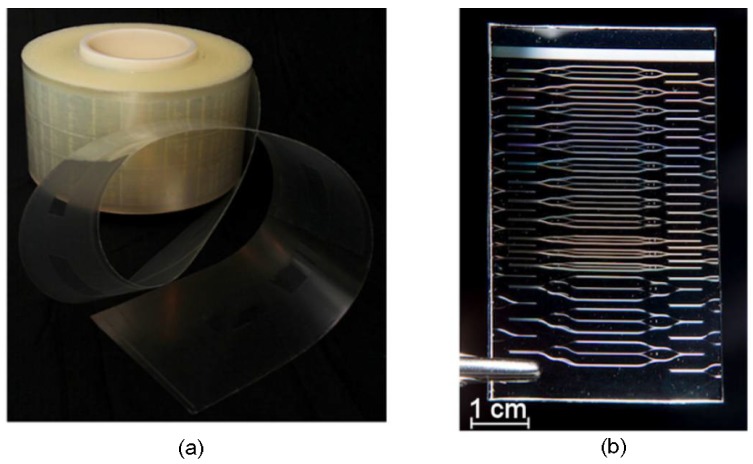
(**a**) Roll of the manufactured sensor by roll-to-roll processing and (**b**) sensor chip cut from the roll [92]. Copyright 2016 OSA.

In order to improve the sensitivity of YI-based photonic biosensors, the slot waveguide can be utilized to replace the channel waveguide in the sensing arm [91]. To manufacture disposable photonic biosensors with low cost and batch-based fabrication, roll-to-roll (R2R) processing can be utilized. In R2R processing, different structures and materials are patterned onto a continuously moving carrier. As shown in Figure 32, the polymeric single-modal photonic integrated circuits for disposable sensors based on YI have been manufactured on rolls with the length of hundreds of meters [92].

## 7. Conclusions and Outlook

We have reviewed the recent progress of the UV imprinted polymer photonic waveguide devices and their applications. By using the liquid, UV curable and spin-coating properties of polymer materials, various passive functional photonic integrated devices, such as microring resonators, waveguide splitters, arrayed waveguide gratings, long-period gratings, and microlenses, can be fabricated using the UV imprinting technique with a simple and low-cost process. With the high TO coefficient, TO-tunable active devices including the microring resonator filters, waveguide Bragg grating filters, optical switches, and variable optical attenuators can be realized with low power consumption. By virtue of the ultrahigh EO coefficient, polymer-based EO modulators can be prepared with high performance. Photonic biosensors based on imprinted polymer waveguide devices can achieve the label-free detection of biomolecules due to the biocompatibility of polymer film.

This manuscript mainly focuses on the polymer photonic integrated devices using the UV imprinting technique, which shows the easy process, low cost, and mass production of polymer waveguides. Certainly, other imprinting-based techniques exerting the advantages of polymer materials, such as thermal imprinting for big dimension structures [93,94], injection molding for arrayed elements [95,96], and ink-jet printing for complex and 3D structures [97,98], can also be utilized to fabricate polymer waveguides. Furthermore, by utilizing the compatibility of polymer materials with diverse materials, such as the III-V semiconductor, the silicon-on-insulator (SOI), silica, and the silicon nitride, hybrid photonic integration can be implemented on polymer platforms. In the common motherboard, individual components with high performance from the respective best-suited material are assembled, yielding great optimization and high cost-efficiency. The hybrid photonic integrated modules can be applied not only in data centers, metro-area optical networks, and DWDM optical networks [99], but also in the lab-on-chip biosensing systems [100,101].
